# Evolution radiologique inhabituelle d'une masse surrénalienne !

**DOI:** 10.11604/pamj.2014.17.291.4228

**Published:** 2014-04-17

**Authors:** Raja El Latifi, Nawal El Ansari

**Affiliations:** 1 Service d'Endocrinologie, Diabétologie, Maladies métaboliques et Nutrition, Laboratoire de recherche PCIM, Faculté de Médecine et de Pharmacie, Université Cadi Ayyad, Marrakech, Maroc

**Keywords:** Masse surrénalienne, radiologie, lymphome, Adrenal mass, radiology, lymphoma

## Image en medicine

La maladie d'Addison est la cause la plus fréquente d'Insuffisance Surrénalienne (IS) primitive acquise. L'aspect radiologique décrit est celui d'une atrophie des surrénales. Elle se traduit initialement par une infiltration lymphocytaire de la corticosurrénale conduisant à une destruction du parenchyme. L'IS se manifeste cliniquement lorsque la rétraction corticale intéresse plus de 90% du cortex, et donc se traduit radiologiquement par une atrophie des glandes qui sont non visualisées. Nous rapportons une observation de maladie d'Addison dont nous suivons l’évolution scanographique : R.T, 24 ans, sans antécédents, admise dans un tableau d'Insuffisance Surrénalienne Lente (ISL) fait de mélanodermie évoluant dans un contexte d'altération de l'etat general, avec déshydratation, vomissements, douleurs abdominales, hypoglycémie, et hypotension à 80/50mmHg. A l'ionogramme une hyponatrémie et hyperkaliémie. La cortisolémie de 8h à 237nmol/l (VN : 270- 556nmol/l). Le bilan de tuberculose et VIH négatifs. Les Anticorps anti 21 hydroxylase très élevés à 5232U/ml (VN< 4U/ml). Les autres atteintes de Poly-endocrinopathie auto-immune non trouvées. La TDM surrénalienne montrait un nodule droit de 42mm × 17mm associé à une infiltration de la loge gauche et des adénopathies infra centimétriques rétro péritonéales (A). Un lymphome surrénalien est suspecté. En attente d'une biopsie, la patiente a été mise sous hydrocortisone avec bonne évolution clinicobiologique spectaculaire. Le contrôle scanographique réalisée avant la ponction (2 mois après) ne montre plus de lésion biopsiable (B). L'image est alors celle d'une infiltration lymphoplasmocytaire de la glande avec nodularité, et passage secondaire à la fibrose et l'atrophie.

**Figure 1:**
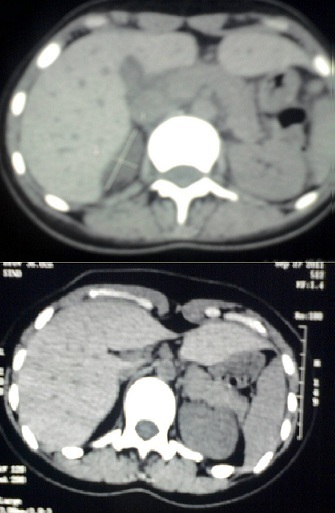
A) TDM en coupe axiale (avec protocole surrénalien) montrant l'infiltration du côté gauche, et le nodule à droite; B) TDM en coupe axiale montrant la disparition des lésions (contrôle après 2 mois)

